# LncRNA AFAP1-AS1 is a prognostic biomarker and serves as oncogenic role in retinoblastoma

**DOI:** 10.1042/BSR20180384

**Published:** 2018-05-15

**Authors:** Fengqin Hao, Yanan Mou, Laixia Zhang, Shuna Wang, Yang Yang

**Affiliations:** 1 Clinical Medical College, Weifang Medical University, No. 7166 Baotong Road West, Weifang 261053, Shandong, China; 2 Department of Ophthalmology, Affiliated Hospital of Weifang Medical University, No. 2428 Yuhe Road, Weifang 261031, Shandong, China

**Keywords:** AFAP1-AS1, biomarkers, large intervening non-coding RNA, oncogene, retinoblastoma

## Abstract

The actin filament-associated protein 1 antisense RNA 1 (AFAP1-AS1) has been found to serve as an oncogenic long noncoding RNA (lncRNA) in most types of human cancer. The role of AFAP1-AS1 in retinoblastoma remains unknown. The purpose of the present study is to explore the clinical significance and biological function of AFAP1-AS1 in retinoblastoma. Levels of AFAP1-AS1 expression were measured in retinoblastoma tissues and cell lines. Loss-of-function study was performed to observe the effects of AFAP1-AS1 on retinoblastoma cell proliferation, cell cycle, migration, and invasion. In our results, AFAP1-AS1 expression was elevated in retinoblastoma tissues and cell lines, and associated with tumor size, choroidal invasion, and optic nerve invasion. Moreover, high expression of AFAP1-AS1 was an independent unfavorable prognostic factor in retinoblastoma patients. The experiment *in vitro* suggested down-regulation of AFAP1-AS1 inhibited retinoblastoma cell proliferation, migration and invasion, and blocked cell cycle. In conclusion, AFAP1-AS1 functions as an oncogenic lncRNA in retinoblastoma.

## Introduction

Retinoblastoma has been reported to be a most common intraocular malignant neoplasm in children [1]. Retinoblastoma accounts for 3% of all childhood cancers and has an incidence of 1 in 14,000–20,000 live births worldwide [2]. Most retinoblastoma patients tend to present with poor prognosis due to lack of early diagnosis and effective therapies, especially in developing country [3,4]. Although retinoblastoma is an uncommon cancer in children, retinoblastoma remains a severe threat to the health of children worldwide, and has garnered a great deal of attention of both clinicians and basic scientists [5].

Long noncoding RNAs (lncRNAs) are a group of noncoding RNA with more than 200 nucleotides in length [6]. Recently, more and more lncRNAs have been suggested to be involved in multiple physiological and pathological processes [7,8]. The actin filament-associated protein 1 antisense RNA 1 (AFAP1-AS1) has been found overexpressed in most types of human cancer tissues and cell lines such as lung cancer, colorectal cancer, gastric cancer, hepatocellular carcinoma, esophageal cancer, pancreatic cancer, ovarian cancer, cholangiocarcinoma, and nasopharyngeal carcinoma [9]. Moreover, a systematic review and meta-analysis showed AFAP1-AS1 high-expression was correlated with TNM stage, lymph node metastasis, and distant metastasis in different types of human tumors [10]. Furthermore, AFAP1-AS1 has been considered as a potential novel biomarker for indicating the clinical outcomes in a total of 1017 cancer patients from eight studies [11].

Although the expression pattern and biological function of AFAP1-AS1 have been reported in several types of human cancers, the role of AFAP1-AS1 in retinoblastoma remains unknown. The purpose of this study is to explore the clinical significance of AFAP1-AS1 in retinoblastoma patient and the biological function of AFAP1-AS1 in retinoblastoma cell.

## Materials and methods

### Patients and samples

The Research Ethics Committees of Affiliated Hospital of Weifang Medical University approved this protocol and written informed consents were obtained from each patient. The entire study was performed based on the Declaration of Helsinki. Fifty-eight freshly frozen retinoblastoma tissue specimens and ten non-cancerous retina tissue specimens were collected from Affiliated Hospital of Weifang Medical University. All clinical specimens were obtained at the time of diagnosis before any antitumor therapy and immediately preserved in liquid nitrogen.

### RNA extraction and quantitative real-time PCR

The total RNA from cell or tissue was extracted from cells with RNAiso Plus (Takara, Japan) and then was reversely transcribed for cDNA by PrimeScript® RT reagent Kit (TaKaRa, Japan). Quantitative real-time PCR was performed using SYBR® Premix Ex TaqTM II (TaKaRa, Japan) on a LightCycler® (Roche, U.S.A.). Relative expression of AFAP1-AS1 was calculated via the comparative cycle threshold (*C*_t_) method and was normalized to the expression of GAPDH. The following primers were used for AFAP1-AS1 and GAPDH: AFAP1-AS1 forward, 5′-TCGCTCAATGGAGTGACGGCA-3′; reverse, 5′-CGGCTGAGACCGCTGAGAACT-3′ and GAPDH forward, 5′-GGAGCGAGATCCCTCCAAAAT-3′; reverse, 5′-GGCTGTTGTCATACTTCTCATGG-3′.

### Cell culture

The human retinoblastoma cell lines (Weri-Rb1 and Y79), ARPE-19 (human retinal pigment epithelial cell line) and human retinal microvascular endothelial cells (HRMECs) were purchased from the Shanghai Institute of Biochemistry and Cell Biology of the Chinese Academy of Sciences. Weri-Rb1 and Y79 were cultured with RPMI 1640 medium (Gibco, U.S.A.) and supplemented with 10% fetal bovine serum (FBS, Gibco, U.S.A.). ARPE-19 was maintained in DMEM medium (Gibco, U.S.A.) with 10% FBS (Gibco, U.S.A.). HRMECs were maintained in EGM-2 SingleQuot Kit Suppl. & Growth Factors (Lonza, U.S.A.). All cell lines were incubated in a humidified chamber at 37°C with 5% CO_2_.

### Cell transfection

The small interfering RNA (siRNA) was synthesized from RiboBio (China). The sequence of AFAP1-AS1 siRNA and control was used as follows: siRNA-AFAP1-AS1: 5′-AACACCAATCCCAAGAGGTGA-3′ and siRNA-NC: 5′-TTCTCCGAACGTGTCACGT-3′. Weri-Rb1 and Y79 cells were transfected using lipofectamine 3000 reagent (Invitrogen, U.S.A.) in Opti-MEM (Gibco, U.S.A.), according to the manufacturer’s instructions. The transfection efficiency was tested by qRT-PCR.

### Cell proliferation assay

Cell proliferation was analyzed using CCK-8 kit (Dojindo, Japan). Briefly, 1 × 10^3^ cells were seeded into 96-well plates with quintuplicate repeat for each condition. At the indicated time points (1, 2, 3, and 4 days), the medium was replaced by 100 μl of fresh culture medium, and 10 μl of CCK-8 solution was added to each well. Plates were incubated for 4 h at 37°C. The absorbance value (OD) was detected at 450 nm.

### Cell cycle analysis

To determine cell cycle distribution, Weri-Rb1 and Y79 cells were plated in six-well plates (4 × 10^5^ cells/well) transfected with siRNA-AFAP1-AS1 or siRNA-NC. After 48 h, cells were harvested and washed with cold phosphate buffer saline (PBS) and fixed with 70% ice-cold ethanol overnight. Fixed cells were rinsed with cold PBS followed by incubation with PBS containing 10 mg/ml propidium iodide and 0.5 mg/ml RNase A for 30 min at 37°C. Cell cycle distribution was then quantified by FACS cytometry assay (BD Biosciences, U.S.A.). The percentage of the cells in G_0_–G_1_, S, and G_2_–M phases were counted and compared.

### Cell migration and invasion assays

In brief, 3 × 10^4^ cells were seeded into a 24-well plate with 8.0 μm pore diameter polycarbonate membrane insert in a transwell apparatus (Corning, U.S.A.). Each sample had three replicates. The lower chamber contained 10% FBS medium as chemoattractant. Cells were incubated for 24 h, Giemsa-stained cells adhering to the lower surface were counted under a microscope. For the cell invasion assay, transwell apparatus was coated with 50% Matrigel (BD Biosciences, U.S.A.).

### Statistical analysis

All data were analyzed for statistical significance using SPSS 17.0 software or GraphPad Prism 5.0 software. The chi-square test was used to analyze the association between AFAP1-AS1 expression and clinicopathological characteristics in retinoblastoma. Two-tailed Student’s *t* test was used for comparisons of two independent groups. Kaplan–Meier method and log-rank test were used to conduct survival analysis. Univariate and multivariate Cox regression models were used to calculate hazard ratios and identify independence of prognostic factor. The significance of survival variables in univariate analyses was included into the final multivariable Cox proportional hazards model. A *P*-value of less than 0.05 was considered statistically significant.

## Results

### AFAP1-AS1 expression is increased in retinoblastoma tissue and cell

**Figure 1:**
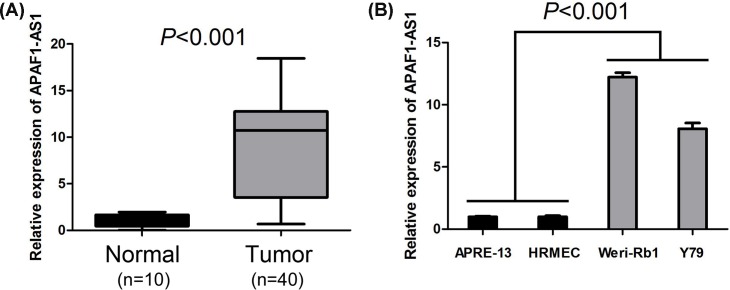
AFAP1-AS1 expression is increased in retinoblastoma tissues and cell lines (**A**) AFAP1-AS1 expression was elevated in retinoblastoma tissue specimens compared with retina tissue specimens. (**B**) AFAP1-AS1 expression was increased in retinoblastoma cell lines compared with normal retina cell lines.

In order to investigate the expression of AFAP1-AS1 in retinoblastoma, the expression level of AFAP1-AS1 was detected by qRT-PCR in retinoblastoma tissue specimens, noncancerous retina tissue specimens, human retinoblastoma cell lines (Weri-Rb1 and Y79), ARPE-19 (human retinal pigment epithelial cell line), and HRMECs. Compared with retina tissue specimens, levels of AFAP1-AS1 expression were significantly elevated in retinoblastoma tissue specimens (*P*<0.001, Figure 1A). Meanwhile, AFAP1-AS1 expression was also increased in retinoblastoma cell lines compared with normal retina cell lines (*P*<0.001, Figure 1B).

### AFAP1-AS1 expression is correlated with clinicopathological features of retinoblastoma patient

We further investigated the correlation between AFAP1-AS1 expression and clinicopathological features of retinoblastoma patients. All retinoblastoma patient cases were divided into AFAP1-AS1 low-expression group and AFAP1-AS1 high-expression group according to published study [12,13]. The correlation between AFAP1-AS1 expression and clinicopathological features was suggested in Table 1. There was no obvious association of AFAP1-AS1 expression with patients’ age (*P*=0.599), gender (*P*=0.791), laterality (*P*=1.000), and pathologic grade (*P*=0.408). However, AFAP1-AS1 expression was closely associated with tumor size (≤10 mm vs >10 mm, *P*=0.030), choroidal invasion (absent vs present, *P*=0.008), and optic nerve invasion (absent vs present, *P*=0.016).

**Table 1:** Correlations between lncRNA AFAP1-AS1 and clinicopathological characteristics in retinoblastoma

Characteristics	*n*	LncRNA AFAP1-AS1 expression		*P*
		High (%)	Low(%)	
Age (years)				
≤14	30	14 (46.7)	16 (53.3)	0.599
>14	28	15 (53.6)	13 (46.4)	
Gender				
Male	33	17 (51.5)	16 (48.5)	0.791
Female	25	12 (48.0)	13 (52.0)	
Size				
≤10 mm	22	7 (31.8)	15 (68.2)	0.030
>10 mm	36	22 (61.1)	14 (38.9)	
Choroidal invasion				
Absent	34	12 (35.3)	22 (67.7)	0.008
Present	24	17 (70.8)	7 (29.2)	
Optic nerve invasion				
Absent	35	13 (37.1)	22 (62.9)	0.016
Present	23	16 (69.6)	7 (30.4)	
Laterality				
Unilateral	44	22 (50.0)	22 (50.0)	1.000
Bilateral	14	7 (50.0)	7 (50.0)	
Pathologic grade				
Well differentiated	20	8 (40.0)	12 (60.0)	0.408
Poorly differentiated	38	21 (55.3)	17 (44.7)	

### AFAP1-AS1 high-expression predicts poor prognosis of retinoblastoma patient

**Figure 2:**
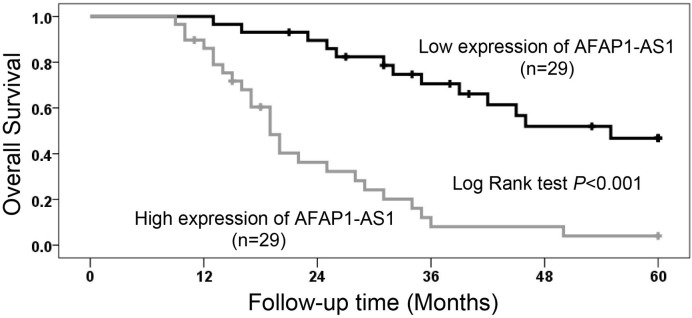
The prognostic value of AFAP1-AS1 expression in retinoblastoma patients AFAP1-AS1 expression was negatively associated with retinoblastoma patients’ overall survival.

In order to investigate the prognostic significance of AFAP1-AS1 expression in retinoblastoma, the correlation between AFAP1-AS1 expression and overall survival time in 58 retinoblastoma cases was analyzed through Kaplan–Meier analysis and log-rank test. We found levels of AFAP1-AS1 expression were markedly associated with retinoblastoma patients’ overall survival, as retinoblastoma patients with AFAP1-AS1 high-expression had shorter survival than those with AFAP1-AS1 low-expression (*P*<0.001, Figure 2). Regardless of large tumor size, present choroidal invasion and present optic nerve invasion, AFAP1-AS1 high-expression was also an unfavorable prognostic factor in retinoblastoma patients based on univariate Cox regression analyses (*P*<0.001, Table 2). In addition, the result of multivariate Cox regression analyses further showed AFAP1-AS1 high-expression was an independent unfavorable prognostic factor in retinoblastoma patients (hazard ratio, HR = 3.598; 95%CI: 1.332–9.718; *P*=0.012, Table 2).

**Table 2:** Univariate and multivariate Cox regression of prognostic factors for overall survival in retinoblastoma

Parameter	Univariate analysis			Multivariate analysis		
	HR	95%CI	*P*	HR	95%CI	*P*
Age (years)	0.876	0.463–1.659	0.685			
≤14 vs >14						
Gender	0.932	0.486–1.788	0.833			
Male vs Female						
Size	3.468	1.531–7.857	0.003	1.203	0.406–3.569	0.739
≤10 mm vs >10 mm						
Choroidal invasion	4.237	2.077–8.640	<0.001	1.750	0.733–4.182	0.208
Absent vs Present						
Optic nerve invasion	2.759	1.436–5.301	0.002	2.001	0.951–-4.210	0.068
Absent vs Present						
Laterality	1.177	0.567–2.441	0.662			
Unilateral vs Bilateral						
Pathologic grade	0.744	0.387–-1.431	0.376			
Well vs Poor						
LncRNA AFAP1-AS1	5.173	2.566–10.429	<0.001	3.598	1.332–9.718	0.012
Low vs High						

Abbreviations: 95% CI, 95% confidence interval; HR, hazard ratio

### Knocking down AFAP1-AS1 expression suppresses retinoblastoma cell proliferation and blocks cell cycle progression

**Figure 3:**
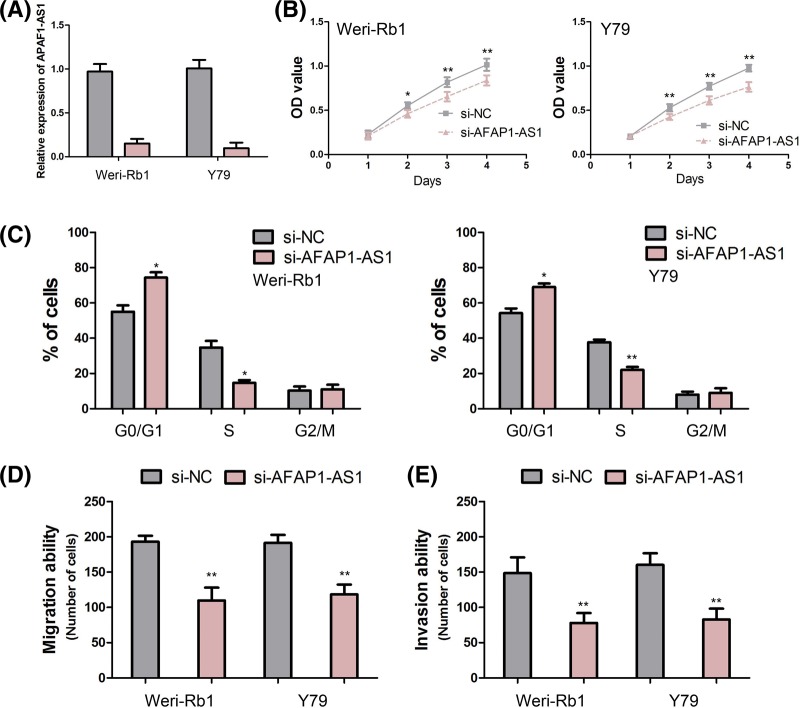
The biological function of AFAP1-AS1 in retinoblastoma (**A**) The efficiency of siRNA-AFAP1-AS1 is confirmed by qRT-PCR in Weri-Rb1 and Y79 cells. (**B**) Knocking down AFAP1-AS1 expression suppresses retinoblastoma cell proliferation. (**C**) Knocking down AFAP1-AS1 expression reduces the percentage of S-phase cells and raised the percentage of G_0_/G_1_-phase cells. (**D**) Knocking down AFAP1-AS1 expression inhibits Weri-Rb1 and Y79 cells migration. (**E**) Knocking down AFAP1-AS1 expression promotes Weri-Rb1 and Y79 cells invasion (*, *P*<0.05; **, *P*<0.001).

In order to investigate the biological functions of AFAP1-AS1 in retinoblastoma cell, we performed loss-of-function study in retinoblastoma cell lines. We knocked down AFAP1-AS1 expression in Weri-Rb1 and Y79 cells through siRNA-AFAP1-AS1, and these efficiencies were confirmed by qRT-PCR (Figure 3A).

The effect of AFAP1-AS1 expression on the retinoblastoma cell proliferation was detected by using CCK-8 assay. The growth curves detected by CCK-8 assay indicated that knocking down AFAP1-AS1 expression significantly suppressed the proliferation of Weri-Rb1 and Y79 cells at 48, 72, and 96 h after transfection (all *P*<0.05, Figure 3B). Furthermore, the results of cell cycle analysis suggested that knocking down AFAP1-AS1 expression significantly reduced the percentage of S-phase cells and raised the percentage of G_0_/G_1_-phase cells (all *P*<0.05, Figure 3C).

### Knocking down AFAP1-AS1 expression inhibits retinoblastoma cell migration and invasion

The effect of AFAP1-AS1 expression on the retinoblastoma cell migration and invasion was measured through cell migration and invasion assays. The results indicated that knocking down AFAP1-AS1 expression obviously inhibited Weri-Rb1 and Y79 cells migration (both *P*<0.001, Figure 3D). Consistent with the results of cell migration assay, the results of invasion assay suggested that knocking down AFAP1-AS1 expression strikingly promoted Weri-Rb1 and Y79 cells invasion (both *P*<0.001, Figure 3E).

## Discussion

Recent decades, AFAP1-AS1 has been found to be overexpressed in most types of human cancers, such as lung cancer [14,15], hepatocellular carcinoma [16,17], cholangiocarcinoma [18,19], colorectal cancer [20,21], gastric cancer [22,23], pancreatic cancer [24], esophageal squamous cell carcinoma [25], renal cell carcinoma [26], gallbladder cancer [27], ovarian cancer [28], tongue squamous cell carcinoma [29], nasopharyngeal carcinoma [30], and thyroid cancer [31]. However, the expression pattern of AFAP1-AS1 in retinoblastoma was still unknown. Thus, we detected the expression of AFAP1-AS1 in retinoblastoma tissues and cell lines, and found levels of AFAP1-AS1 expression were significantly elevated in retinoblastoma tissues and cell lines compared with normal retina tissue and retina cell lines respectively. Meanwhile, we further investigate the clinical significance of AFAP1-AS1 in retinoblastoma cases through analyzing the correlation between AFAP1-AS1 expression and clinicopathological features. We observed that AFAP1-AS1 high-expression was closely associated with large tumor size, presence of choroidal invasion, and optic nerve invasion. In lung cancer, Deng et al. [32] found high levels of AFAP1-AS1 expression in tumor tissues were associated with clinical stage, smoking history, infiltration degree, lymph node metastasis, and distant metastasis. Then, Li et al. [33] reported that high serum AFAP1-AS1 expression levels were also correlated with present distant metastasis, present lymph node metastasis, poor clinical stage, and larger tumor size in lung cancer patients. Moreover, Wang et al. [34] showed AFAP1-AS1 overexpression was closely associated with tumor size, TNM stage, and distant metastasis in colorectal cancer patients. In pancreatic ductal adenocarcinoma, Ye et al. [35] suggested that high levels of AFAP1-AS1 were correlated with lymph node metastasis and perineural invasion, but Fu et al. [36] showed that AFAP1-AS1 expression was only associated with tumor size. The discrepancy between Ye et al.’s data and Fu et al.’s data would be most likely due to the different samples and the limited sample number in Fu et al.’s study. In addition, the significance of AFAP1-AS1 was also explored in hepatocellular carcinoma [16], cholangiocarcinoma [19], gastric cancer [23], esophageal squamous cell carcinoma [25], gallbladder cancer [27], ovarian cancer [28], tongue squamous cell carcinoma [29], and nasopharyngeal carcinoma [37].

More and more studies showed AFAP1-AS1 acts as an important prognostic factor for human cancers. Liu et al. [11] performed meta-analysis including a total of 1017 cancer patients from eight studies, and found cancer patients with high AFAP1-AS1 expression had a shorter recurrence-free survival, a worse progression-free survival, and a poorer overall survival than those with low AFAP1-AS1 expression. Furthermore, Wang et al. [10] also conducted meta-analysis based on the literature and GEO datasets, and presented more evidence that AFAP1-AS1 served as a biomarker predicting tumor progression, prognosis, and lymph node metastasis in human cancers. However, the correlation between AFAP1-AS1 expression and the survival of retinoblastoma patients has been seldom reported. In our study, we found AFAP1-AS1 high-expression was significantly associated with short overall survival and acted as an independent unfavorable prognostic factor in retinoblastoma patients, which is consistent with the prognostic value of AFAP1-AS1 in other cancers.

AFAP1-AS1 has been suggested to function as an oncogenic lncRNA in cancer progression. Zhang et al. [16] reported that down-regulation of AFAP1-AS1 expression suppressed hepatocellular carcinoma cell growth and metastasis *in vitro* and *in vivo*, promoted cell apoptosis, and blocked cell cycle in S-phase through modulating RhoA/Rac2 signaling. Similarly, Ye et al. [35] showed down-regulation of AFAP1-AS1 expression resulted in the inhibition of cell proliferation, migration, and invasion, and up-regulation of AFAP1-AS1 expression induced cell proliferation, migration, and invasion in pancreatic ductal adenocarcinoma. In nasopharyngeal carcinoma, Bo et al. [30] demonstrated that knockdown of AFAP1-AS1obviously reduced cell migration and invasive capability, but had no effect on cell proliferation. The effect of AFAP1-AS1 on the retinoblastoma cell proliferation, migration, and invasion was still unknown. We performed loss-of-function study in retinoblastoma cell lines, and found knocking down AFAP1-AS1 expression inhibited retinoblastoma cell proliferation, migration and invasion, and blocked cell cycle.

The genomic context showed AFAP1-AS1 is localized at the antisense chain of the gene coding AFAP1 protein, and there are overlapping and complementary regions between the second AFAP1-AS1 exon and AFAP1 exons 14, 15, and 16. In lung cancer, Zeng et al. [38] indicated down-regulation of AFAP1-AS1 elevated AFAP1 protein expression and modulated some molecules correlated with Rho/Rac GTPase family members and actin cytokeratin signaling pathway. Moreover, AFAP1-AS1 also has been found to modulate AFAP1 translation or increase the half-life of AFAP1 protein, but that AFAP1-AS1 did not affect AFAP1 transcription in nasopharyngeal carcinoma and cholangiocarcinoma cells [18,30]. In our following studies, we will explore the association between AFAP1-AS1 and AFAP1 in retinoblastoma cell.

In conclusion, AFAP1-AS1 is up-regulated in retinoblastoma tissues and cell lines, and associated with clinicopathological features including prognosis in retinoblastoma patients. Down-regulation of AFAP1-AS1 inhibits retinoblastoma cell proliferation, migration and invasion, and blocks cell cycle.

## Abbreviations

AFAP1-AS1: actin filament-associated protein 1 antisense RNA 1
HRMEC: human retinal microvascular endothelial cells
LncRNA: long noncoding RNA
TNM: tumor, node, metastes
